# Komplette Visuserholung nach Nd:YAG-Laserpolitur der Kunstlinsenvorderfläche

**DOI:** 10.1007/s00347-021-01373-w

**Published:** 2021-04-15

**Authors:** Adrien Quintin, Berthold Seitz, Tim Berger, Alaa Din Abdin

**Affiliations:** Klinik für Augenheilkunde, Universitätsklinikum des Saarlandes (UKS), Homburg/Saar, Deutschland

## Hintergrund

Im Rahmen einer Kataraktoperation kann die Vorderfläche der Intraokularlinse (IOL) unter Umständen an der Spaltlampe eingetrübt erscheinen. Diese Präzipitate der Kunstlinsenvorderfläche können bei chronischen entzündlichen Prozessen konfluieren und verdichten, sodass eine beträchtliche Visusminderung verursacht wird [1]. Bei konfluierenden dichten Ablagerungen, die trotz Verwendung topischer Steroide persistieren und mit einer Visusminderung einhergehen, stellt die Neodymium-Yttrium-Aluminium-Garnet(Nd:YAG)-Laserpolitur der Kunstlinsenvorderfläche immer noch eine minimal-invasive, effektive und relativ sichere therapeutische Alternative dar.

## Anamnese und Befund

**Figure 1:**
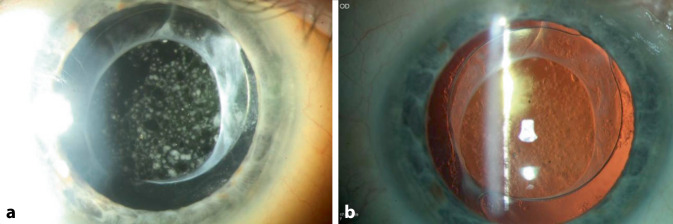

**Figure 2:**
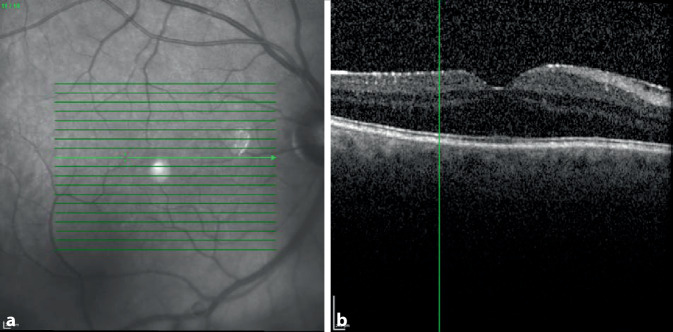

Wir berichten über einen 67-jährigen kaukasischen Patienten, der sich mit Beschlägen auf der Kunstlinsenvorderfläche am rechten Auge vorstellte. Die Vorstellung erfolgte 24 Monate nach der Durchführung einer komplikationslosen Phakoemulsifikation mit Implantation einer Hinterkammerlinse (HOYA Vivinex XY1 [Hoya Surgical Optics, Tokyo, Japan], monofokal hydrophobes Acryl). Anamnestisch klagte der Patient über eine Visusminderung am betroffenen Auge. Der bestkorrigierte Dezimalvisus am betroffenen rechten Auge betrug vor der Kataraktoperation cc 0,5pp und 1 Monat postoperativ sc 1,0. Der Patient stellte sich 8 Monate postoperativ notfallmäßig aufgrund einer Uveitis intermedia am rechten Auge vor, wobei die bestkorrigierte Sehschärfe rechts cc 1,0pp und links cc 1,0 betrug. Es erfolgte eine stationäre Aufnahme zur erweiterten Uveitisabklärung (Thoraxröntgenaufnahme ohne pathologischen Befund, Lues negativ, Borrelien negativ, regelrechter MRT-Befund des Schädels, ACE regelrecht, IL‑2 regelrecht) sowie zur Einleitung einer lokalen und systemischen Steroidtherapie. Bei der poststationären Kontrolle nach 4 Wochen betrug die Sehschärfe an beiden Augen 1,0 wie auch bei einer erneuten notfallmäßigen Vorstellung nach 2 Monaten im Rahmen eines Rezidivs der Uveitis intermedia. Der bestkorrigierte Dezimalvisus am betroffenen rechten Auge betrug bei der Vorstellung 24 Monate postoperativ ccs 0,2p und am linken Auge ccs 1,0. Klinisch zeigten sich am betroffenen Auge ausgeprägte weißliche Beschläge auf der Vorderfläche der Intraokularlinsenoptik (Abb. 1a, b) bei einem ansonsten reizfreien vorderen Augenabschnitt. Trotz eines reduzierten Einblickes bestanden fundoskopisch keine Hinweise eines Uveitisrezidivs. Eine zirkuläre Netzhautanlage konnte sonographisch gesichert werden. Die optische Kohärenztomographie der Makula zeigte eine trockene Netzhaut mit geringer nichttraktiver fokaler epiretinaler Gliose bei regelrechter Netzhautdicke (Abb. 2). Am Partnerauge zeigte sich eine Cataracta corticonuclearis incipiens. Der applanatorisch gemessene Augeninnendruck war beidseits im Normbereich. Außer einem Tränenersatzmittel bestand keine weitere ophthalmologische Lokaltherapie. Die Allgemeinanamnese ergab eine koronare Herzkrankheit, arterielle Hypertonie sowie einen Diabetes mellitus Typ 2.

## Therapie und Verlauf

**Figure 3:**
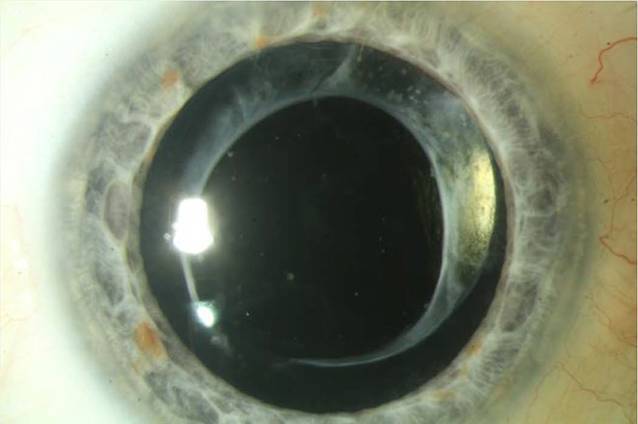

Zur Behandlung der ausgeprägten Visusminderung entschied man sich aufgrund der minimalen Invasivität einer Laserbehandlung für eine Nd:YAG-Laserpolitur der Kunstlinsenvorderfläche (VisuLas® YAG III, Zeiss, Oberkochen, Deutschland). Insgesamt wurden 69 Laserherde mit einer Energie von 1,5 mJ (Wellenlänge 1064 nm, Pulsdauer < 4 ns) appliziert. Die Laserpulse wurden diskret vor die IOL-Optik fokussiert mit einer Defokussierung von −150 µm (aufgrund möglicher fester Fokussierungseinstellungen am Gerät: „ant.“ = −150 µm; „0“ = ohne Defokussierung; „post.“ = +150 µm). Der Patient wurde anschließend mit Prednisolonacetat Augentropfen 8‑mal täglich therapiert, welche wöchentlich um 1 Tropfen reduziert wurden. Die Kontrolluntersuchung am nächsten Tag zeigte bereits eine komplette Visuserholung auf ccs 1,0p am gelaserten Auge bei reizfreier Vorderkammer mit klarem Kunstlinsenzentrum ohne Linsenpits auf der Linsenvorderfläche (Abb. 3). Der applanatorisch gemessene Augeninnendruck am gelaserten Auge betrug 24 mm Hg, sodass eine Therapieergänzung mit Brimonidin-Augentropfen 2‑mal täglich empfohlen wurde.

## Diskussion

Im Zuge einer Kataraktoperation kann die Vorderfläche der Intraokularlinse (IOL) unter Umständen an der Spaltlampe „eingetrübt“ erscheinen. Diese Trübungen können unter anderem durch Blutungen, entzündliche Ablagerungen („Präzipitate“), Bildung einer fibrinösen Pupillenmembran, Schrumpfung einer kleinen anterioren Kapselöffnung nach der Kapsulorhexis [8] oder durch Linsenverkalkungen nach sekundären intraokularen Operationen verursacht werden, insbesondere bei intraokularer Gaseingabe (z. B. nach posteriorer lamellärer Keratoplastik oder nach Pars-plana-Vitrektomie) [2, 4, 7]. Präzipitate, bestehend aus Pigment, Entzündungszellen, Fibrin, Blutabbauprodukten und anderen Elementen, können sich auf der Rückfläche der Hornhaut sowie auf der Kunstlinsenvorderfläche bilden, welche unmittelbar im postoperativen Zeitraum auftreten [1]. Diese Präzipitate bilden sich häufig unter Verwendung lokaler Steroide zurück. Im Rahmen einer chronischen Entzündung, wie beispielsweise einer Uveitis, oder einer intraokularen Blutung können diese Präzipitate jedoch konfluieren und ausreichend dicht werden, sodass eine beträchtliche Visusminderung hervorgerufen wird [1].

Bereits vor 30 Jahren hatten Brauweiler und Ohrloff die Effektivität einer Nd:YAG-Laserpolitur bei Beschlägen der Kunstlinsenvorderfläche beschrieben [3, 5]. Zu den möglichen Komplikationen dieser minimal-invasiven Laserbehandlung zählen Augeninnendruckerhöhungen sowie Kunstlinsenbeschädigungen durch Laser-induzierte Linseneinschläge, sog. „Pits“. In der Regel stellen diese für den Patienten jedoch keine visuelle Beeinträchtigung dar. Um das Risiko eines Linsenschadens zu minimieren, sollte die Nd:YAG-Laserpolitur nach dem Prinzip von „niedriger Energie und Defokussierung“ erfolgen, wobei der Laserfokus nicht auf die Präzipitate, sondern direkt vor diesen fokussiert sein sollte [6]. Nach der Laserbehandlung sollten topische Steroide verwendet werden, um einer intraokulären Entzündung des vorderen Augenabschnittes entgegenzuwirken. Bei sorgfältiger Anwendung hat sich die Nd:YAG-Laserpolitur als effektive und relativ sichere Therapie erwiesen, um Beschläge der Kunstlinsenvorderfläche erfolgreich und dauerhaft therapieren zu können. In vielen Fällen kann dadurch eine invasivere Operation wie eine chirurgische Politur oder womöglich ein IOL-Austausch erspart werden.

## Fazit für die Praxis

- Zarte Präzipitate auf der Kunstlinsenvorderfläche verschwinden postoperativ häufig spontan, ggf. mithilfe der Verwendung von topischen Steroiden.
- Bei persistierenden und konfluierenden dichten Ablagerungen, die den Visus beeinflussen, bildet die Nd:YAG-Laserpolitur der Kunstlinsenvorderfläche eine minimal-invasive und effektive therapeutische Alternative und gilt als relativ sicher bei sorgfältiger Anwendung nach dem Prinzip von „niedriger Energie und Defokussierung“.
